# Long Noncoding RNA TCONS_00016406 Attenuates Lipopolysaccharide-Induced Acute Kidney Injury by Regulating the miR-687/PTEN Pathway

**DOI:** 10.3389/fphys.2020.00622

**Published:** 2020-06-18

**Authors:** Xuelan Liu, Na Zhu, Bo Zhang, Shao Bo Xu

**Affiliations:** Department of Emergency, Ningbo Medical Center Li Huili Hospital, Ningbo, China

**Keywords:** long noncoding RNA 6406, miR-687, PTEN, sepsis, acute kidney injury

## Abstract

Acute kidney injury (AKI) is a common and serious complication of sepsis accompanied by kidney dysfunction resulting from various etiologies and pathophysiological processes. Unfortunately, there is currently no ideal therapeutic strategy for AKI. Numerous studies have confirmed that long noncoding RNAs (lncRNAs) play important regulatory roles in the pathogenesis of sepsis-associated AKI. In this study, lncRNA TCONS_00016406 (termed lncRNA 6406), a novel lncRNA identified by using TargetScan, was significantly downregulated in the kidney tissues of mice with sepsis-associated AKI. This study aimed to explore the role of lncRNA 6406 in lipopolysaccharide (LPS)-induced AKI and its potential molecular mechanism. The models of sepsis-induced AKI (called LPS-induced AKI models) in mice and cell lines were established with male C57BL/6 mice and renal tubular epithelial (PTEC) cells, respectively. Twenty-four hours after LPS administration, kidneys and cell samples were collected after various treatments to examine the alterations in the lncRNA 6406 levels and to evaluate the effects on LPS-induced inflammation, oxidative stress, and apoptosis through real-time PCR (RT-PCR) analysis, western blotting, and terminal deoxynucleotidyl transferase-mediated dUTP nick end labeling (TUNEL) staining. The results revealed that lncRNA 6406 could significantly attenuate LPS-induced AKI, as shown by the alleviation of inflammation, the suppression of oxidative stress and the inhibition of apoptosis. Mechanistically, a luciferase reporter assay and additional research showed that lncRNA 6406 functioned as a ceRNA to sponge miRNA-687, thereby modulating LPS-stimulated AKI by targeting the miR-687/PTEN axis; thus, this study presents a novel therapeutic strategy or sepsis-associated AKI.

## Introduction

Sepsis is a kind of systemic inflammatory response syndrome caused by an infection (Girardot et al., 2019; Jiang et al., 2019b; Lin et al., 2019). Acute kidney injury (AKI), a severe complication of sepsis, can trigger the occurrence of organ dysfunction, thereby leading to high morbidity and mortality in most patients with septic shock (Gong et al., 2019). The morbidity of septic AKI caused by endotoxemia is only 3–5% in general patients, but reaches nearly 50% in patients in intensive care units (ICUs; Shi et al., 2019; Wiersema et al., 2019). It has been confirmed by some studies that the pathogenesis of sepsis-induced AKI is highly associated with renal hemodynamic abnormalities and inflammatory responses (Wiersema et al., 2019). Unfortunately, the underlying mechanisms of sepsis-induced AKI remain unclear. Hence, providing novel insight into the pathogenesis of AKI is extremely crucial for the development of potential therapies for AKI.

Increasing evidence has considered that a variety of effector cells, inflammation, oxidative stress, and apoptosis play key roles in the process of endotoxemia-induced AKI (Islam et al., 2019). Inflammation can induce the occurrence of apoptosis and necrosis and the activation of a series of inflammatory cells in patients with endotoxic inflammation. These factors not only initiate the immune inflammatory response but also stimulate the oxidative stress response through the oxygen free radicals produced during the process of injury, thus aggravating renal tubular injury (Islam et al., 2019; Jiang et al., 2019a). Therefore, improving the levels of inflammation, oxidative stress, and apoptosis in patients with endotoxemia and renal injury will be highly beneficial to AKI patients.

Long noncoding RNAs (lncRNAs) are defined as noncoding RNAs with a length of approximately 200 nucleotides, and these molecules were previously described as “junk” transcripts (Zhang et al., 2017b). In fact, lncRNAs are expressed not only in the nucleus but also in the cytoplasm. Many studies have demonstrated that lncRNAs participate in the regulation of various physiological and pathological processes in diseases by modulating the function of their target miRNAs (Liu et al., 2019). Different expression levels of lncRNAs can be seen circulating in the blood or tissues of patients with different diseases, including tumors, cardiovascular diseases, and nervous system and immune system diseases. Recently, by comparing the RNA and lncRNA expression profiles in the blood of AKI patients, healthy controls, and ischemic disease patients, some studies have found that certain lncRNAs in the blood of AKI patients are dysregulated, and that the level of the endogenous antisense lncRNA TapSAKI in circulation can predict the survival rate of AKI patients (Tian et al., 2019).

In our study, we focused on a novel lncRNA (lncRNA 6406) that was significantly downregulated in AKI patients based on prechip analysis (Chun-Mei et al., 2016). On the basis of a series of *in vitro* and *in vivo* experiments evaluating the inflammatory response, oxidative stress, and apoptosis, we identified that lncRNA 6406 could attenuate sepsis-associated AKI by modulating the miR-687/PTEN signaling pathway, which may provide a new therapeutic approach for AKI in the future.

## Materials and Methods

### Animal Experiments

This study was approved by the Ethics Committee of University. All the experiments conformed to all the relevant regulatory standards. Male C57BL/6 mice were obtained from Cavens Lab Animal (Changzhou, China) and housed in a specific pathogen-free (SPF) laboratory animal facility under a 12-h light/dark cycle; the mice were given free access to standard chow and water. To induce septic AKI, the mice were intraperitoneally injected with lipopolysaccharide (LPS) from *Escherichia coli* O111:B4 (5 mg/kg) (Cat No. L2630, Sigma-Aldrich, USA) to induce sepsis. Twelve hours after the injection, the mice were sacrificed, and kidney samples were fixed in 4% paraformaldehyde (PFA) or snap frozen in azote nitrite and stored at −80°C for further analysis. Three weeks prior to the LPS injection, 100 μl of a kidney-targeting recombinant adeno-associated virus carrying an overexpression-LncRNA TCONS_00016406 construct or a cntl-LncRNA construct (Hanbio Biotechnology Co., China) was administered to the mice *via* tail vein injection. These procedures were followed by further experiments.

### Cell Culture and Treatments

BU.MPT cells, a mouse kidney proximal tubular epithelial cell line (PTEC) was purchased from the Cell Bank of the Chinese Academy of Sciences, were cultured in Dulbecco’s modified Eagle’s medium (Gibco, USA) containing 10% fetal bovine serum at 37°C in an incubator with an atmosphere of 5% CO_2_. To identify suitable concentrations and time points, the PTEC cells were treated with different concentrations of LPS (0, 1, 2, 5, and 10 μg/ml) for 24 h and with 2 μg/ml LPS for different time points (0, 6, 12, 24, and 48 h). To evaluate the effect of lncRNA 6406 on LPS-treated PTEC cells, the lncRNA 6406 overexpression plasmid was constructed, and a shRNA targeting lncRNA 6406 (Sh-LncRNA 6406) was obtained from Gene Pharma Co., Ltd. LncRNA 6406 (2 μg/ml) or Sh-LncRNA 6406 (2 μg/ml) was transfected into the PTEC cells prior to LPS exposure. To further investigate the mechanism by which lncRNA 6406 exerts its effect, the PTEC cells were cotransfected with a miR-687 mimic (50 nM) or PTEN siRNA (75 nM) followed by LPS treatment. All transfections were performed using Lipo-3000 (Invitrogen, USA) according to the manufacturer’s instructions.

### Cell Counting Kit-8 (CCK-8) Assay

PTEC cell viability was detected using the Cell Counting Kit-8 (CCK-8; Dojindo, Japan). After all the treatments, 10 μl of 5 mg/ml CCK-8 reagent was added to each well, and then, the plate was incubated at 37°C for 2 h. A microplate reader (Thermo Fisher, USA) was used to measure the absorbance at 490 nm.

### Real-Time Polymerase Chain Reaction (RT-PCR) Analysis

RNA samples were obtained from whole cell lysate or specific subcellular fractions using a PARISTM Kit (Applied Biosystem, USA), and cDNA was synthesized using the iScript™ cDNA Synthesis Kit (Bio-Rad, USA). Q-PCR was performed using SYBR Green (Applied Biosystems, USA) on a Roche LightCycler 480 PCR System according to the manufacturer’s instructions. The relative expression levels of the relevant genes were compared with the respective internal control using the 2^−△△Ct^ method. The primers used in the qRT-PCR are shown in Supplementary Table 1.

### Western Blotting Analysis

Western blots were performed using standard procedures. Briefly, cells or tissues were lysed with RIPA buffer (Beyotime, China). Equal quantities of the total proteins were separated by SDS-PAGE gels, transferred to PVDF membranes, and blocked with 5% BSA for 2 h. The membranes were incubated with specific primary antibodies at 4°C overnight, including antibodies against GAPDH (Cat. No. 10494-1-AP, Proteintech, China), BAX (Cat. No. 50599-2-Ig, Proteintech, China), and BCL2 (Cat. No. 26593-1-AP, Proteintech, China). The membranes were then incubated with the corresponding secondary antibodies for 2 h at room temperature. The protein bands were visualized using an enhanced chemiluminescence (ECL) kit in a ChemiDoc XRS Plus luminescent image analyzer (Bio-Rad, USA), and the expression of each protein was analyzed using ImageJ software after normalization to its internal control.

### Luciferase Reporter Assay

To generate the LncRNA 6406 wt-luc vector, a fragment of the 3′ UTR of LncRNA 6406 that contained the target site of miR-687 was obtained *via* PCR amplification and subsequently cloned into the pGL3-Basic Vector (Promega, Madison, WI, USA). The MutaBest kit (Takara, Tokyo, Japan) was then used to generate the LncRNA 6406 mutant-luc vector. Forty-eight hours after transfection, the luciferase activities were examined by using a dual luciferase reporter assay system (RiboBio, China).

### TUNEL Staining

Apoptosis of the PTEC cells or frozen kidney sections was examined using a TUNEL kit (Roche, Germany) according to the manufacturer’s instructions. Briefly, the samples were fixed with 4% PFA, permeabilized with 0.5% Triton X-100 in PBS, and blocked with 5% bovine serum album (BSA). Then, the cells or tissue sections (5 μm) were stained with the TUNEL Apoptosis Detection Kit (Roche, Germany) and counterstained with DAPI. After staining, the images were captured by a fluorescence microscope system (Carl Zeiss AG, Germany).

### Renal Function Analysis

To assess renal function, mouse blood samples were collected before the mice were sacrificed, and the concentrations of blood urea nitrogen (BUN) and serum creatinine (SCR) were measured using available kits (Thermo Fisher Scientific, USA).

### Histological Analysis

For the morphometric analyses, transverse tissue sections (5 μm) of formalin-fixed and paraffin-embedded murine kidney tissues were stained with a standard hematoxylin and eosin (H&E) procedure. LUCIA software (Nikon) was used to capture the images.

Dihydroethidium (DHE, Cat No. S0063, Beyotime, China) was used to examine reactive oxygen species (ROS). Briefly, transverse tissue sections (5 μm) were subjected to DHE staining according to the manufacturer’s instructions, and the images were captured by a fluorescence microscope system (Carl Zeiss AG, Germany).

### Statistical Analysis

All the experimental data were analyzed using GraphPad Prism software 6.0 and data represent the mean ± SD of three independent measurements. An independent-sample *t*-test was used for two-group comparisons, and one-way ANOVA followed by Bonferroni’s *post hoc* test was used for multiple-group comparisons; *p* < 0.05 was considered statistically significant.

## Results

### LncRNA 6406 Is Downregulated in the Kidneys of Mice in the LPS-Induced AKI Model

**Figure 1:**
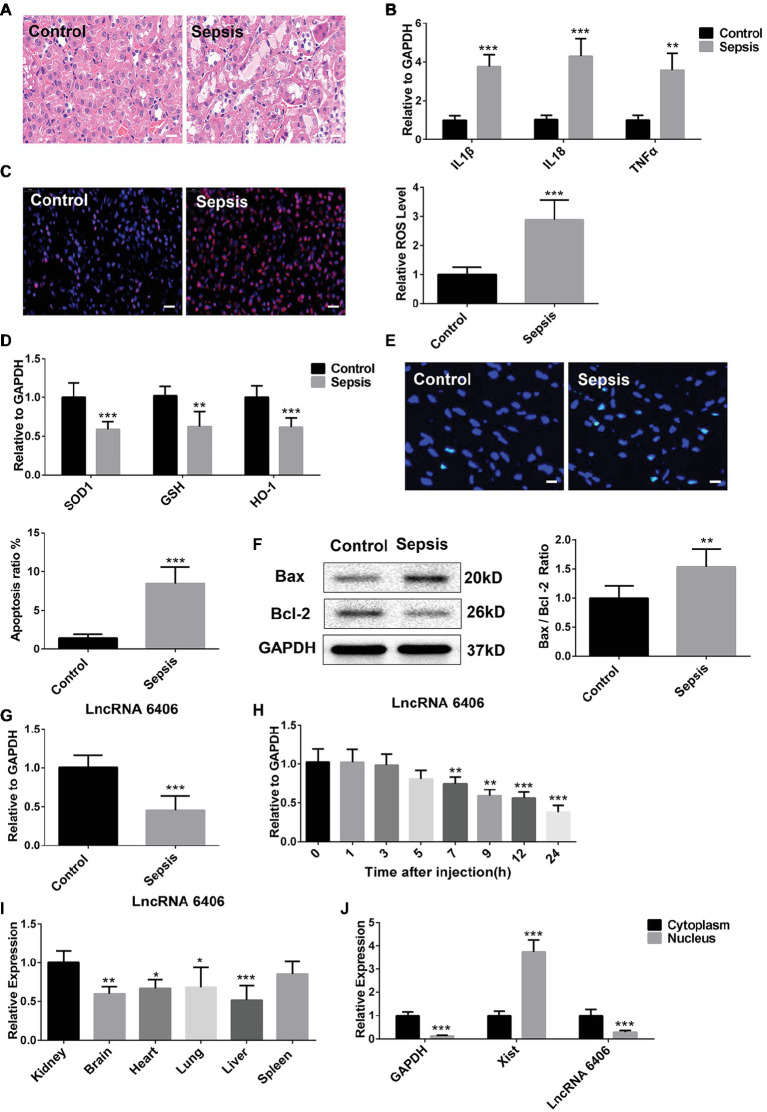
Downregulation of long noncoding RNAs (lncRNA) 6406 was observed in the kidneys of mice in the lipopolysaccharide (LPS)-induced acute kidney injury (AKI) model. (**A**) HE staining was used to observe the outline of renal morphology, scale bar = 20 μm. (**B**) The relative expression of inflammatory cytokines in the kidneys of mice in the LPS-induced AKI model was detected by real-time PCR (RT-PCR) analysis (*n* = 6). (**C**,**D**) The LPS-induced oxidative stress in the kidneys of mice in the AKI model was determined by Dihydroethidium (DHE) staining and RT-PCR analysis (*n* = 6), scale bar = 20 μm. (**E**,**F**) The TUNEL-positive cells and Bax and Bcl-2 protein expression levels in the LPS-induced AKI model were determined to evaluate apoptosis (*n* = 6), scale bar = 10 μm. (**G**) The relative expression of lncRNA 6406 was detected in the AKI mouse kidneys (*n* = 6). (**H**) The expression of lncRNA 6406 was confirmed at different time points after LPS injection in the mice (*n* = 6). (**I**) The expression level of LncRNA 6406 was determined in different tissues (*n* = 6). (**J**) The subcellular localization of lncRNA 6406 was detected by extracting specific subcellular fractions from the PTEC cell line (*n* = 6). Each experiment was conducted independently three times. ^*^p < 0.05; ^**^p < 0.01; ^***^p < 0.001; versus respective control.

To explore whether LncRNA 6406 played a functional role in sepsis-related AKI, we first constructed a mouse model of LPS-induced renal injury and determined the levels of inflammation, oxidative stress, and apoptosis in the renal tissues of the control and LPS groups. The HE staining results showed that in the LPS group, there was punctate and granular inflammatory cell infiltration in the renal cortex and interstitium, swelling and vacuolar degeneration in most renal tubular epithelial cells, and falling off of the brush-like edge (Figure 1A). The levels of inflammatory cytokines, including IL-1β, TNF-α, and IL-18, in the kidneys of the mice in the LPS-induced AKI group were obviously upregulated compared with those in the control groups, confirming the activation of the inflammatory response by LPS injection (Figure 1B). The DHE staining and real-time PCR (RT-PCR) results revealed that the level of oxidative stress in kidney tissues of the LPS group was significantly increased compared with that of the control group (Figures 1C,D). In addition, to provide more evidence supporting the successful establishment of the LPS-induced AKI model, the level of LPS-induced cell apoptosis was examined by TUNEL assay, and the TUNEL staining results showed that the number of apoptotic cells (green) was clearly increased in the kidneys of the LPS-challenged mice (Figure 1E). Consistently, the changes in the expression of apoptosis-related proteins (including the Bax and Bcl-2 proteins) were consistent with the above mentioned results. As demonstrated in Figure 1F, the expression level of the Bax protein was significantly increased, while the Bcl-2 proteins exhibited the opposite trend. In addition, the ratio of the Bax/Bcl-2 expression level was elevated to a certain extent. Taken together, these results suggested that the successful establishment of the LPS-induced AKI model presented evidence of increased inflammatory responses, oxidative stress, and apoptosis in the kidney tissues of these mice. Notably, the expression of lncRNA 6406 in the kidneys of the mice in the LPS-induced AKI group was remarkably decreased and time-dependent (from 7 to 24 h) compared with that in the control group (Figures 1G,H). Finally, we verified the expression of lncRNA 6406 in different tissues and its subcellular localization in the cells. The RT-PCR analysis results showed that the expression level of lncRNA 6406 in the kidney was significantly higher than that in the heart, brain, lung, and liver (Figure 1I), and that it was mainly expressed in the cytoplasm (Figure 1J). These data suggest that lncRNA 6406 may have a potential function in sepsis-induced AKI.

### Downregulation of LncRNA 6406 Is Observed in the LPS-Stimulated *in vitro* Model

**Figure 2:**
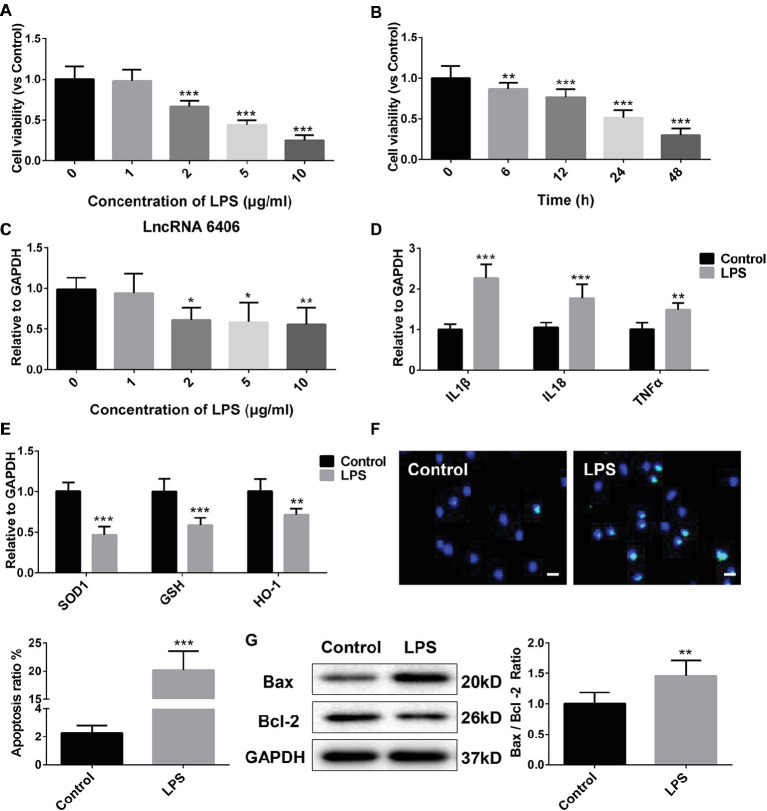
The downregulation of LncRNA 6406 was determined in an *in vitro* cell model of LPS-induced AKI. (**A,B**) The viability of PTEC cells was evaluated after exposure to different concentrations of LPS (0, 1, 2, 5, and 10 μg/ml) for 24 h and after exposure to 2 μg/ml LPS for different time points (0, 6, 12, 24, and 48 h) (*n* = 6). (**C**) The relative expression was determined after exposure to LPS at different concentrations (*n* = 6). (**D**) The relative expression of inflammatory cytokines was detected in the LPS-stimulated AKI cell model. (**E**) Oxidative stress in the PTEC cells was determined after LPS stimulation by RT-PCR analysis (*n* = 6). (**F**,**G**) Apoptosis in the PTEC cells were evaluated *via* TUNEL assay and western blot analysis (*n* = 6), scale bar = 10 μm. Each experiment was conducted independently three times. ^*^p < 0.05; ^**^p < 0.01; ^***^p < 0.001; versus respective control.

To choose the most appropriate LPS concentration and to confirm the expression of lncRNA 6406 in LPS-treated PTEC cells, the cell viability of PTEC cells treated with a series of LPS concentrations (0, 1, 2, 5, and 10 μg/ml) for different time points (0, 6, 12, 24, and 48 h) was assessed using a CCK-8 assay. As illustrated in Figures 2A,B, the PTEC cell viability gradually decreased with increasing LPS concentrations, and the cell viability significantly decreased once the LPS concentration reached 2 μg/ml. To further validate the effect of 2 μg/ml LPS, the viability of PTEC cells treated with LPS (2 μg/ml) for different time points (0, 6, 12, 24, and 48 h) was measured, and the results showed that the PTEC cell viability was gradually decreased with time, which was consistent with the aforementioned results. In addition, we found that LPS (2 μg/ml) significantly reduced the lncRNA 6406 expression, but with the increase in LPS concentration, the lncRNA 6406 expression did not change further (Figure 2C). Therefore, we chose 2 μg/ml as the final concentration of LPS *in vitro*. As shown in Figure 2D, the levels of inflammatory cytokines, including IL-1β, TNF-α, and IL-18, in the LPS-induced AKI cell group were also obviously upregulated compared with those in the control groups, which was consistent with the inflammatory response exhibited in Figure 1B. Additionally, a parallel experiment measuring the SOD1, GSH, and HO-1 levels in PTEC cells was performed to confirm that LPS administration induced the oxidative stress. As shown in Figure 2E, the SOD1, GSH, and HO-1 levels in the PTEC cells were all downregulated to varying extents upon LPS treatment. Next, the successful establishment of a cell model of LPS-stimulated AKI was confirmed by the promotion of apoptosis in the PTEC cells, as determined by the results of TUNEL staining and an enhanced ratio of Bax/Bcl-2 (Figures 2F,G).

### LncRNA 6406 Attenuates LPS-Stimulated AKI by Mitigating Cell Inflammation, Oxidative Stress, and Apoptosis

**Figure 3:**
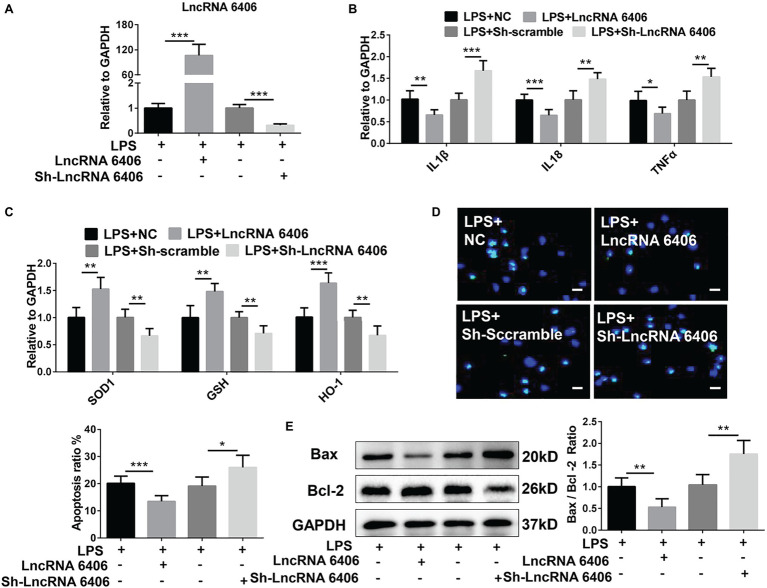
The function of lncRNA 6406 in LPS-stimulated AKI was determined. (**A**) The transfection efficacies of the lncRNA 6406 overexpressing vector (pcDNA-LncRNA 6406) or knockdown vector (Sh-LncRNA 6406) were verified in the PTEC cells *via* RT-PCR assays (*n* = 6). (**B**,**C**) LncRNA 6406 overexpression inhibited the LPS-induced upregulation of pro-inflammatory cytokines (including IL-1β, TNF-α, and IL-8) and oxidative stress-related proteins, while lncRNA 6406 knockdown showed the opposite effect on the PTEC cells (*n* = 6). (**D**,**E**) LncRNA 6406 attenuated LPS-induced apoptosis, and lncRNA 6406 inhibition further promoted LPS-induced apoptosis *in vitro* (*n* = 6), scale bar = 10 μm. Each experiment was conducted independently three times. ^*^p < 0.05; ^**^p < 0.01; ^***^p < 0.001; versus respective control.

To test the function of lncRNA 6406 in the pathogenesis of sepsis-associated AKI, lncRNA 6406 overexpression (pcDNA-LncRNA 6406) and inhibition vectors (Sh-LncRNA 6406) were transfected into PTEC cells. As shown in Figure 3A, the transfection efficacies were successfully confirmed by the corresponding changes in the lncRNA 6406 levels. To determine whether lncRNA 6406 can alleviate LPS-stimulated AKI, pcDNA-LncRNA 6406, and Sh-LncRNA 6406 were transfected into PTEC cells, followed by LPS administration. As shown in Figure 3B, lncRNA 6406 overexpression inhibited the upregulation of pro-inflammatory cytokines (including IL-1β, TNF-α, and IL-18) induced by LPS treatment. By contrast, the downregulation of lncRNA 6406 by sh-lncRNA 6406 exhibited the opposite effect on the pro-inflammatory cytokine production, which was consistent with the above results. As shown in Figure 3C, lncRNA 6406 overexpression alleviated AKI *via* oxidative stress suppression, as shown by the drastically elevated levels of SOD1, GSH, and HO-1 in the PTEC cells; however, lncRNA 6406 knockdown led to the opposite trend. As expected, the LPS-induced increase in apoptosis was decreased by the upregulation of lncRNA 6406, but exacerbated by the knockdown of lncRNA 6406 (Figures 3D,E). In summary, these results revealed that lncRNA 6406 possesses protective effects against LPS-induced cell damage by modulating inflammation, oxidative stress, and apoptosis.

### LncRNA 6406 Relives LPS-Induced Cell Damage by Sponging miR-687

**Figure 4:**
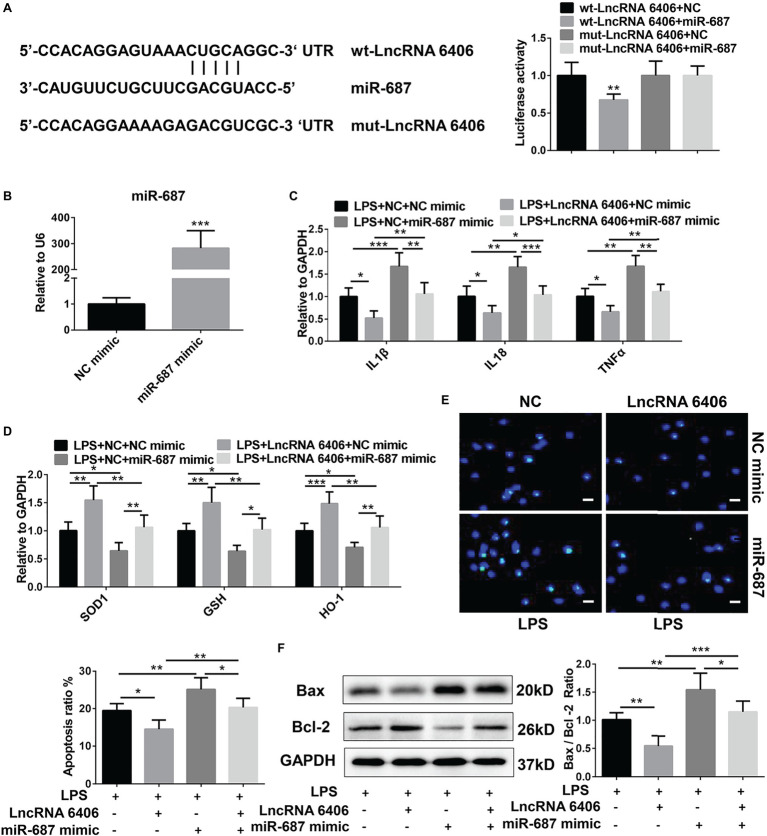
MiR-687 negatively regulated the function of lncRNA 6406. (**A**) The luciferase reporter assay indicated that miR-687 is a target of lncRNA 6406. (**B**) The transfection efficacy of the miR-687 mimic was determined by RT-PCR analysis (*n* = 6). (**C**,**D**) The miR-687 mimic inhibited the lncRNA 6406 overexpression-induced functional changes in the inflammatory response and oxidative stress (*n* = 6). (**E**,**F**) The miR-687 mimic promoted LPS-induced apoptosis and inhibited the effect of lncRNA 6406 on apoptosis in LPS-stimulated PTEC cells (*n* = 6), scale bar = 10 μm. Each experiment was conducted independently three times. ^*^p < 0.05; ^**^p < 0.01; ^***^p < 0.001; versus respective control.

Accumulating evidence has revealed that lncRNAs and miRNAs are highly involved in the modulation of biological processes in various diseases. To further explore the possible mechanism of lncRNA 6406 in the pathogenesis of sepsis-associated AKI, bioinformatics software (TargetScan) was applied to predict the miRNAs that might interact with lncRNA 6406. Here, miR-646, which possesses a lncRNA 6406 binding site as described, was identified as a candidate, and the interaction between lncRNA 6406 and miR-646 was verified. As a result, as shown in Figure 4A, wild-type lncRNA 6406 (wt-lncRNA 6406) and mutant lncRNA 6406 (mut-lncRNA 6406) reporter vectors were constructed to conduct a luciferase reporter assay, and the results indicated that lncRNA 6406 could combine with miR-687. To further examine whether miR-687 is involved in the role of lncRNA 6406 in LPS-induced cell injury, we used the lncRNA 6406 overexpression plasmid and an miRNA mimic (miR-687 mimic) to transfect cells separately or jointly; the transfection efficiency of the miR-687 mimic is shown in Figure 4B. Consequently, as shown in Figures 4C–F, the corresponding RT-PCR, TUNEL staining, and western blotting analyses revealed that the protective effects of lncRNA 6406 on LPS-induced cell injury, including the amelioration of inflammation, oxidative stress, and apoptosis, were drastically reversed by miR-687 overexpression.

### LncRNA 6406 Ameliorates LPS-Induced AKI by Modulating PTEN

**Figure 5:**
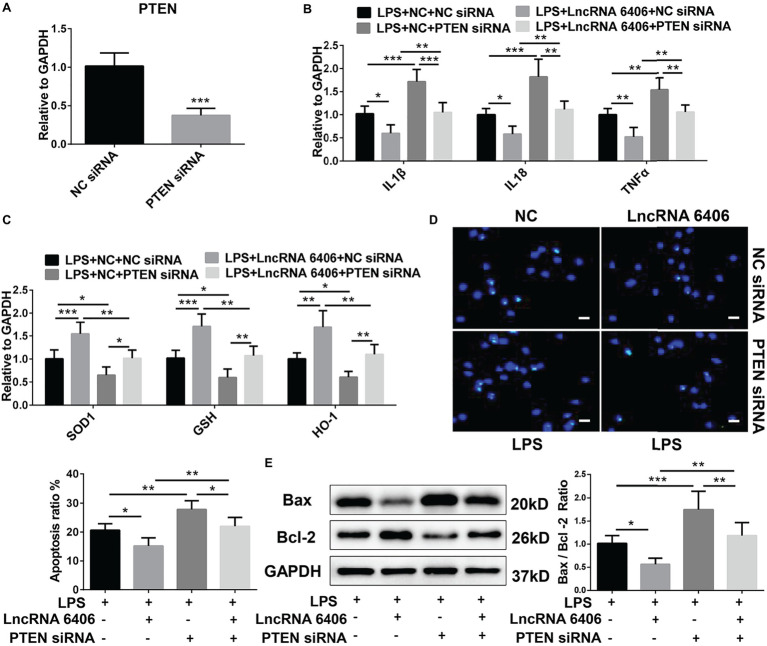
LncRNA 6406 exerted its effect on AKI by regulating PTEN. (**A**) RT-PCR analysis revealed the efficacy of the PTEN siRNA (*n* = 6). (**B**,**C**) The inhibition of PTEN inhibited the effect of lncRNA 6406 on inflammation and oxidative stress. (**D**,**E**) The inhibition of PTEN increased the LPS-induced apoptosis and attenuated the effect of lncRNA 6406 in PTEC cells (*n* = 6), scale bar = 10 μm. Each experiment was conducted independently three times. ^*^p < 0.05; ^**^p < 0.01; ^***^p < 0.001; versus respective control.

PTEN, as a downstream target gene of miR-687, is involved in the pathogenesis of AKI by regulating apoptosis and inflammation (Wang et al., 2011; Palliyaguru et al., 2016; Schaalan and Mohamed, 2016; Luan et al., 2017). Herein, to determine whether lncRNA 6406 could ameliorate LPS-induced AKI by modulating the PTEN signaling pathway, we first constructed PTEN siRNA and verified its efficiency (Figure 5A); then, we transfected cells separately or jointly with lncRNA 6406 overexpression plasmids. The RT-PCR analysis results confirmed that the LPS-induced upregulation of pro-inflammatory cytokines (including IL-1β, TNF-α, and IL-18) and suppression of oxidative stress-related proteins (SOD1, GSH, and HO-1) were further exaggerated by PTEN knockdown and that the inhibition of PTEN partially reversed the function of lncRNA 6406 (Figures 5B,C). Consistently, the TUNEL staining and western blotting analysis results showed that the apoptosis aggravated by PTEN downregulation could be rescued by lncRNA 6406 overexpression (Figures 5D,E). These data indicate that lncRNA 6406 attenuates LPS-induced cell injury by regulating PTEN expression.

### LncRNA 6406 Suppresses LPS-Induced AKI *in vivo*

**Figure 6:**
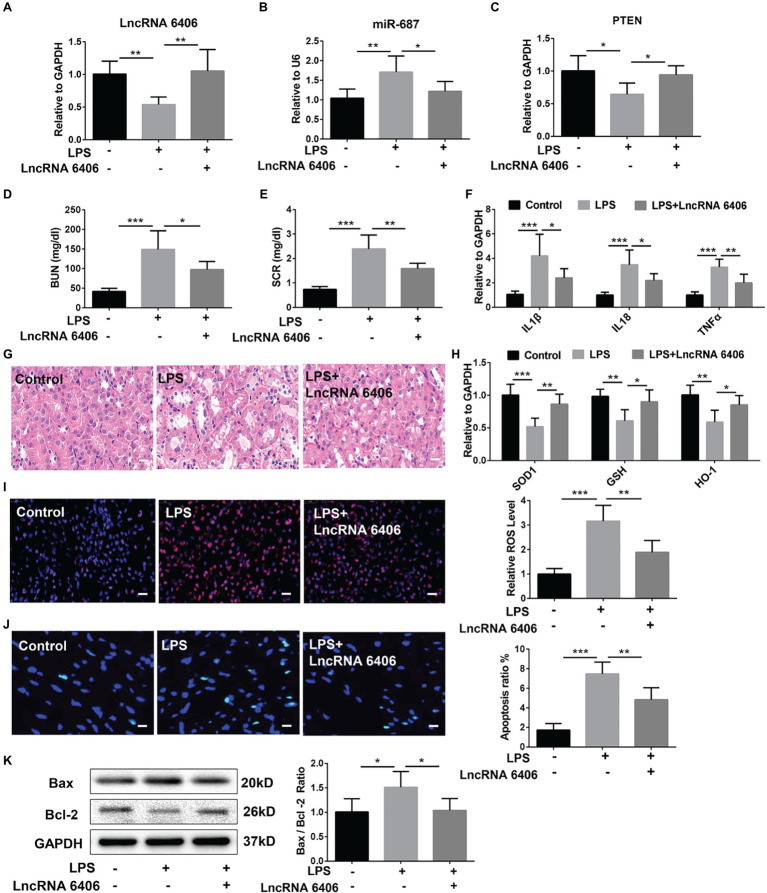
LncRNA 6406 protected against LPS-induced AKI *in vivo*. (**A**–**C**) The relative expression of lncRNA 6406, miR-687 and PTEN was examined *via* qRT-PCR analysis (*n* = 6). (**D**,**E**) The difference in blood urea nitrogen (BUN) and serum creatinine (SCR) were detected *via* ELISA after 24 h of the LPS injection in mice. (**F**) RT-PCR analysis demonstrated that lncRNA 6406 attenuated the LPS-induced inflammatory response in the kidneys of the AKI mice (*n* = 6). (**G**) LncRNA 6406 improved the outline of renal morphology in LPS-induced AKI, scale bar = 20 μm. (**H**,**I**) RT-PCR analysis and DHE staining revealed that lncRNA 6406 improved the LPS-induced oxidative stress in the kidney (*n* = 6), scale bar = 20 μm. (**J**,**K**) LncRNA 6406 decreased the LPS-induced apoptosis in the kidney *in vivo* (*n* = 6), scale bar = 10 μm. Each experiment was conducted independently three times. ^*^p < 0.05; ^**^p < 0.01; ^***^p < 0.001; versus respective control.

To further study the effect of lncRNA 6406 on LPS-induced AKI and the role of lncRNA 6406 in regulating miR-687/PTEN *in vivo*, we established an LPS-induced AKI model and overexpressed lncRNA 6406 by AAV9-lncRNA 6406. RT-PCR analysis showed downregulation of the PTEN level and upregulation of the miR-687 level in the mice after LPS injection, and these changes in expression were effectively reversed by lncRNA 6406 overexpression (Figures 6A–C). To elucidate the effect of lncRNA 6406 on LPS-induced renal dysfunction, we also observed that the LPS-induced increase in BUN and SCR in the serum was significantly inhibited by lncRNA 6406 overexpression (Figures 6D,E). RT-PCR analysis results indicated that the expression of pro-inflammatory cytokines (including IL-1β, TNF-α, and IL-18) induced by LPS in mice was significantly decreased after lncRNA 6406 overexpression (Figure 6F). As shown in Figure 6G, the HE staining demonstrated that tubular epithelial cell swelling, brush border loss, cell membrane bleb formation, interstitial edema, cytoplasmic vacuolization, and cell necrosis were observed in the LPS groups, and was alleviated by lncRNA 6406 overexpression. In addition, the RT-PCR and DHE staining results confirmed that the LPS-induced levels of ROS and the related indicators of oxidative stress (SOD1, GSH, and HO-1) were significantly inhibited by the overexpression of lncRNA 6406 (Figures 6H,I). Consistent with the above results, the LPS-induced apoptosis in mice was also efficiently alleviated by lncRNA 6406 overexpression, which was shown by TUNEL staining and a decreased Bax/Bcl-2 ratio (Figures 6J,K).

## Discussion

Sepsis is a systemic inflammatory response syndrome resulting from microbial infection, which is associated with high morbidity and mortality rates. AKI, as a common complication of sepsis, has attracted much attention due to its high mortality rate of up to 75% and its various complications, such as chronic kidney disease (Chawla et al., 2014; Zhang et al., 2017a). Studies have reported that AKI is induced in more than 50% of sepsis patients, and this AKI leads to renal dysfunction and triggers the excessive production of inflammatory cytokines and mediators. It has been confirmed that the pathogenesis of AKI involves multiple aspects, including renal inflammation, ischemia, acute hypoxia, hypercoagulation, oxidative stress, and microcirculatory disturbance, etc., (Shum et al., 2016). However, the detailed mechanisms underlying AKI remain unclear. Hence, it is essential to reveal the pathogenesis of AKI, thereby paving the way for the development of a new, effective therapy for AKI. The participation of pro-inflammatory cytokines or mediators due to the systemic inflammatory response is thought to play a crucial role in the pathogenesis of sepsis-associated AKI. In addition, the excessive production of the relevant pro-inflammatory cytokines may even trigger the occurrence of organ dysfunction, such as kidney injury (Khajevand-Khazaei et al., 2019).

TNF-α, as a critical cytokine, has been shown to exacerbate renal injury by modulating TNF receptors (Cunningham et al., 2002). Changes in TNF-α can also be associated with changes in the concentrations of other relevant inflammatory factors (Kurt et al., 2007). It has been reported that AKI could be attenuated by downregulating TNF-α expression in mice (Faubel et al., 2007). IL-1β and IL-6 were also identified as prognostic indicators for sepsis-associated AKI due to their crucial function in local acute inflammation and acute renal damage (Chawla et al., 2007; Zhao et al., 2016). As shown in this study, the secretion of the above pro-inflammatory cytokines was evidently promoted in renal tissues and PTEC cells after LPS administration. More importantly, the protective effect of lncRNA 6406 on ameliorating LPS-stimulated inflammation was obviously shown by the low levels of pro-inflammatory cytokine production in the *in vivo* and *in vitro* experiments, and this effect strengthened cellular immune responses, activated defensive functions, and prevented the occurrence of infection.

The role of oxidative stress is also highlighted in the pathogenesis of AKI (Agarwal et al., 2016). It has been reported that DEX pretreatment showed antioxidative and renal protective effects that attenuated AKI in mice (Yu et al., 2016). The stress-responsive enzyme heme oxygenase-1 (HO-1) is known to prevent ischemia-reperfusion injury (IRI), which is a main cause of AKI. Hence, we examined the antioxidant activities of SOD1, GSH, and HO-1 in response to various treatments in this study. As shown in the results, the suppression of oxidative stress-related proteins (SOD1, GSH, and HO-1) by LPS treatment was successfully alleviated by lncRNA 6406 overexpression. Moreover, the apoptosis induced by the inflammatory responses in AKI is also essential in the development and progression of AKI. Bax served as a proapoptotic protein, while Bcl-2 functioned as an antiapoptotic factor. The elevated ratio of Bax/Bcl-2, which is a hallmark of exacerbated apoptosis caused by LPS treatment, was markedly downregulated due to lncRNA 6406 overexpression, confirming the protective effect of lncRNA 6406 against AKI. Therefore, there were fewer TUNEL-positive cells in the LPS-induced group treated with lncRNA 6406 overexpression than in the control group.

Mechanistically, lncRNAs serve as ceRNAs to sponge miRNAs. For instance, the lncRNA NEAT1 promotes hypoxia-induced renal tubular epithelial apoptosis by downregulating miR-27a-3p (Jiang et al., 2019a). Jiang et al. also reported that the overexpression of the lncRNA HOTAIR can alleviate AKI in septic rats by inhibiting the apoptosis of kidney tissues by downregulating the miR-34a/Bcl-2 signaling pathway (Jiang et al., 2019b). Herein, to further explore the underlying mechanism of lncRNA 6406 in ameliorating sepsis-associated AKI, we identified miR-687 as a target of lncRNA 6406 on the basis of TargetScan. In addition, the functions of miRNAs (e.g., miR-21, miR-24, miR-30 family, miR-126, miR-127, miR-150, miR-494, and miR-687) and lncRNAs (e.g., TapSAKI, AK139328, and lncRNA-PRINS) in the pathogenesis of AKI have been revealed in research. Consequently, the results showed that lncRNA 6406 could relieve LPS-induced AKI by sponging miR-687, which was consistent with our hypothesis.

PTEN has attracted much more attention due to its crucial function in the pathophysiological processes of acute injury or dysfunction in multiple organs (Ning et al., 2004; Lan et al., 2012; Yang et al., 2016). In addition, PTEN has been emphasized as a central regulator in the development of AKI (Bhatt et al., 2015; Potočnjak and Domitrović, 2016). In the present study, to test whether lncRNA6406 could exert a significant effect on LPS-induced AKI by modulating the PTEN pathway, LPS-induced AKI was exacerbated by PTEN knockdown *via* transfection of PTEN siRNA, and then lncRNA 6406 was administered to assess its interaction with PTEN in the pathogenesis of AKI. Our results suggested that lncRNA6406 could exert a significant effect on LPS-induced AKI by regulating the miR-687/PTEN axis, thus providing deeper insight for the development of a new therapeutic strategy for AKI.

In conclusion, based on the *in vivo* and *in vitro* experiments that aimed to evaluate inflammation, oxidative stress, apoptosis, and renal function, we showed that lncRNA 6406 could attenuate sepsis-associated AKI by modulating the miR-687/PTEN signaling pathway.

## Data Availability Statement

All datasets generated for this study are included in the article/Supplementary Material.

## Ethics Statement

The animal research was reviewed and approved by Ethics Committee of LIHUILI Hospital of Ningbo Medical Center.

## Author Contributions

XL designed and performed the experiments, analyzed data, and wrote the manuscript. NZ and BZ performed the *in vitro* and *in vivo* experiments. SX designed and supervised the study, and performed manuscript editing.

## Conflict of Interest

The authors declare that the research was conducted in the absence of any commercial or financial relationships that could be construed as a potential conflict of interest.

## Supplementary Material

The Supplementary Material for this article can be found online at: https://www.frontiersin.org/articles/10.3389/fphys.2020.00622/full#supplementary-material.

## References

[ref1] Agarwal A. Dong Z. Harris R. Murray P. Parikh S. M. Rosner M. H.. (2016). Cellular and molecular mechanisms of AKI. J. Am. Soc. Nephrol. 27, 1288–1299. 10.1681/ASN.2015070740, PMID: 26860342 PMC4849836

[ref2] Bhatt K. Wei Q. Pabla N. Dong G. Mi Q.-S. Liang M. (2015). MicroRNA-687 induced by hypoxia-inducible factor-1 targets phosphatase and tensin homolog in renal ischemia-reperfusion injury. J. Am. Soc. Nephrol. 26, 1588–1596. 10.1681/ASN.2014050463, PMID: 25587068 PMC4483585

[ref3] Chawla L. S. Eggers P. W. Star R. A. Kimmel P. L. (2014). Acute kidney injury and chronic kidney disease as interconnected syndromes. N. Engl. J. Med. 371, 58–66. 10.1056/NEJMra1214243, PMID: 24988558 PMC9720902

[ref4] Chawla L. S. Seneff M. G. Nelson D. R. Williams M. Levy H. Kimmel P. L.. (2007). Elevated plasma concentrations of IL-6 and elevated APACHE II score predict acute kidney injury in patients with severe sepsis. Clin. J. Am. Soc. Nephrol. 2, 22–30. 10.2215/CJN.02510706, PMID: 17699383

[ref5] Chun-Mei H. Qin-Min G. Shu-Ming P. Xiang-Yang Z. (2016). Expression profiling and ontology analysis of circulating long non-coding RNAs in septic acute kidney injury patients. Clin. Chem. Lab. Med. 54, e395–e399. 10.1515/cclm-2015-1281, PMID: 27341563

[ref6] Cunningham P. N. Dyanov H. M. Park P. Wang J. Newell K. A. Quigg R. J. (2002). Acute renal failure in endotoxemia is caused by TNF acting directly on TNF receptor-1 in kidney. J. Immunol. 168, 5817–5823. 10.4049/jimmunol.168.11.5817, PMID: 12023385

[ref7] Faubel S. Lewis E. C. Reznikov L. Ljubanovic D. Hoke T. S. Somerset H.. (2007). Cisplatin-induced acute renal failure is associated with an increase in the cytokines interleukin (IL)-1beta, IL-18, IL-6, and neutrophil infiltration in the kidney. J. Pharmacol. Exp. Ther. 322, 8–15. 10.1124/jpet.107.119792, PMID: 17400889

[ref8] Girardot T. Schneider A. Rimmelé T. (2019). Blood purification techniques for sepsis and septic AKI. Semin. Nephrol. 39, 505–514. 10.1016/j.semnephrol.2019.06.010, PMID: 31514914

[ref9] Gong Y. Ding F. Zhang F. Gu Y. (2019). Investigate predictive capacity of in-hospital mortality of four severity score systems on critically ill patients with acute kidney injury. J. Investig. Med. 67, 1103–1109. 10.1136/jim-2019-001003, PMID: 31575668 PMC6900215

[ref10] Islam M. S. Miao L. Yu H. Han Z. Sun H. (2019). Ethanol extract of *illicium henryi* attenuates LPS-induced acute kidney injury in mice via regulating inflammation and oxidative stress. Nutrients 11:1412. 10.3390/nu11061412, PMID: 31234591 PMC6627762

[ref11] Jiang X. Li D. Shen W. Shen X. Liu Y. (2019a). LncRNA NEAT1 promotes hypoxia-induced renal tubular epithelial apoptosis through downregulating miR-27a-3p. J. Cell. Biochem. 120, 16273–16282. 10.1002/jcb.28909, PMID: 31090110

[ref12] Jiang Z.-J. Zhang M.-Y. Fan Z.-W. Sun W.-L. Tang Y. (2019b). Influence of lncRNA HOTAIR on acute kidney injury in sepsis rats through regulating miR-34a/Bcl-2 pathway. Eur. Rev. Med. Pharmacol. Sci. 23, 3512–3519. 10.26355/eurrev_201904_17717, PMID: 31081107

[ref13] Khajevand-Khazaei M.-R. Azimi S. Sedighnejad L. Salari S. Ghorbanpour A. Baluchnejadmojarad T.. (2019). S-allyl cysteine protects against lipopolysaccharide-induced acute kidney injury in the C57BL/6 mouse strain: involvement of oxidative stress and inflammation. Int. Immunopharmacol. 69, 19–26. 10.1016/j.intimp.2019.01.026, PMID: 30665040

[ref14] Kurt A. Aygun A. D. Godekmerdan A. Kurt A. Dogan Y. Yilmaz E. (2007). Serum IL-1beta, IL-6, IL-8, and TNF-alpha levels in early diagnosis and management of neonatal sepsis. Mediat. Inflamm. 2007:31397. 10.1155/2007/31397, PMID: 18274637 PMC2220039

[ref15] Lan R. Geng H. Polichnowski A. J. Singha P. K. Saikumar P. McEwen D. G.. (2012). PTEN loss defines a TGF-β-induced tubule phenotype of failed differentiation and JNK signaling during renal fibrosis. Am. J. Physiol. Renal Physiol. 302, F1210–F1223. 10.1152/ajprenal.00660.2011, PMID: 22301622 PMC3362177

[ref16] Lin Z. Jin J. Shan X. (2019). Fish oils protects against cecal ligation and puncture-induced septic acute kidney injury via the regulation of inflammation, oxidative stress and apoptosis, oxidative stress and apoptosis. Int. J. Mol. Med. 44, 1771–1780. 10.3892/ijmm.2019.4337, PMID: 31545434 PMC6777667

[ref17] Liu Y. Wang J. Dong L. Xia L. Zhu H. Li Z.. (2019). Long noncoding RNA HCP5 regulates pancreatic cancer gemcitabine (GEM) resistance by sponging Hsa-miR-214-3p to target HDGF. Onco Targets Ther. 12, 8207–8216. 10.2147/OTT.S222703, PMID: 31632071 PMC6781945

[ref18] Luan Y. Chen M. Zhou L. (2017). MiR-17 targets PTEN and facilitates glial scar formation after spinal cord injuries via the PI3K/Akt/mTOR pathway. Brain Res. Bull. 128, 68–75. 10.1016/j.brainresbull.2016.09.017, PMID: 27693649

[ref19] Ning K. Pei L. Liao M. Liu B. Zhang Y. Jiang W.. (2004). Dual neuroprotective signaling mediated by downregulating two distinct phosphatase activities of PTEN. J. Neurosci. 24, 4052–4060. 10.1523/JNEUROSCI.5449-03.2004, PMID: 15102920 PMC6729419

[ref20] Palliyaguru D. L. Chartoumpekis D. V. Wakabayashi N. Skoko J. J. Yagishita Y. Singh S. V.. (2016). Withaferin a induces Nrf2-dependent protection against liver injury: role of keap1-independent mechanisms. Free Radic. Biol. Med. 101, 116–128. 10.1016/j.freeradbiomed.2016.10.003, PMID: 27717869 PMC5154810

[ref21] Potočnjak I. Domitrović R. (2016). Carvacrol attenuates acute kidney injury induced by cisplatin through suppression of ERK and PI3K/Akt activation. Food Chem. Toxicol. 98, 251–261. 10.1016/j.fct.2016.11.004, PMID: 27825756

[ref22] Schaalan M. Mohamed W. (2016). Determinants of hepcidin levels in sepsis-associated acute kidney injury: impact on pAKT/PTEN pathways? J. Immunotoxicol. 13, 751–757. 10.1080/1547691X.2016.1183733, PMID: 27266727

[ref23] Shi Y. Hua Q. Li N. Zhao M. Cui Y. (2019). Protective effects of evodiamine against LPS-induced acute kidney injury through regulation of ROS-NF-κ B-mediated inflammation. Evid. Based Complement. Alternat. Med. 2019:2190847. 10.1155/2019/2190847, PMID: 30941189 PMC6421037

[ref24] Shum H.-P. Yan W.-W. Chan T. (2016). Recent knowledge on the pathophysiology of septic acute kidney injury: a narrative review. J. Crit. Care 31, 82–89. 10.1016/j.jcrc.2015.09.017, PMID: 26475099

[ref25] Tian X. Ji Y. Liang Y. Zhang J. Guan L. Wang C. (2019). LINC00520 targeting miR-27b-3p regulates OSMR expression level to promote acute kidney injury development through the PI3K/AKT signaling pathway. J. Cell. Physiol. 234, 14221–14233. 10.1002/jcp.28118, PMID: 30684280 PMC13483068

[ref26] Wang Y.-D. Zhang L. Cai G.-Y. Zhang X.-G. Lv Y. Hong Q.. (2011). Fasudil ameliorates rhabdomyolysis-induced acute kidney injury via inhibition of apoptosis. Ren. Fail. 33, 811–818. 10.3109/0886022X.2011.601830, PMID: 21797820

[ref27] Wiersema R. Koeze J. Hiemstra B. Pettilä V. Perner A. Keus F.. (2019). Associations between tricuspid annular plane systolic excursion to reflect right ventricular function and acute kidney injury in critically ill patients: a SICS-I sub-study. Ann. Intensive Care 9:38. 10.1186/s13613-019-0513-z, PMID: 30868290 PMC6419793

[ref28] Yang X. Qin Y. Shao S. Yu Y. Zhang C. Dong H.. (2016). MicroRNA-214 inhibits left ventricular remodeling in an acute myocardial infarction rat model by suppressing cellular apoptosis via the phosphatase and tensin homolog (PTEN). Int. Heart J. 57, 247–250. 10.1536/ihj.15-293, PMID: 26973267

[ref29] Yu X. Chi X. Wu S. Jin Y. Yao H. Wang Y.. (2016). Dexmedetomidine pretreatment attenuates kidney injury and oxidative stress during orthotopic autologous liver transplantation in rats. Oxidative Med. Cell. Longev. 2016:4675817. 10.1155/2016/4675817, PMID: 26682005 PMC4670681

[ref30] Zhang S. Ma J. Sheng L. Zhang D. Chen X. Yang J.. (2017a). Total coumarins from *Hydrangea paniculata* show renal protective effects in lipopolysaccharide-induced acute kidney injury via anti-inflammatory and antioxidant activities. Front. Pharmacol. 8:872. 10.3389/fphar.2017.00872, PMID: 29311915 PMC5735979

[ref31] Zhang L. Yang Z. Trottier J. Barbier O. Wang L. (2017b). Long noncoding RNA MEG3 induces cholestatic liver injury by interaction with PTBP1 to facilitate shp mRNA decay. Hepatology 65, 604–615. 10.1002/hep.28882, PMID: 27770549 PMC5258819

[ref32] Zhao H. Zheng Q. Hu X. Shen H. Li F. (2016). Betulin attenuates kidney injury in septic rats through inhibiting TLR4/NF-κB signaling pathway. Life Sci. 144, 185–193. 10.1016/j.lfs.2015.12.003, PMID: 26656467

