# Supplementary material for: Long Noncoding RNA TCONS_00016406 Attenuates Lipopolysaccharide-Induced Acute Kidney Injury by Regulating the miR-687/PTEN Pathway
Source: Front Physiol. 2020 Jun 18;11:622. doi: 10.3389/fphys.2020.00622 (PMC7325890; doi:10.3389/fphys.2020.00622)
Supplement: Supplementary file 1 [file Data_Sheet_1.docx]

Table 1 List for Primers used for qRT-PCR

| Gene | Species | | Forward Primer | Reverse Primer |
| --- | --- | --- | --- | --- |
| IL1β  IL18  TNFα  SOD1  GSH  HO-1  PTEN  GAPDH | Mouse  Mouse  Mouse  Mouse  Mouse  Mouse  Mouse  Mouse |  | *GCAACGGGAAGATTCTGAAG*  *GGCCGACTTCACTGTACAACCGC*  *ACGGCATGGATCTCAAAGAC*  *AACCAGTTGTGTTGTCAGGAC*  *CAAAGCAGGCCATAGACAGGG*  *AAGCCGAGAATGCTGAGTTCA*  *TGGATTCGACTTAGACTTGACCT*  *CCTT CC GTGT TCCTA CCCC* | *TGACAAACTTCTGCCTGACG*  *TGGTCTGGGGTTCACTGGCACT*  *GTGGGTGAGGAGCACGTAGT*  *CCACCATGTTTCTTAGAGTGAGG*  *AAAAGCGTGAATGGGGCATAC*  *GCCGTGTAGATATGGTACAAGGA*  *GCGGTGTCATAATGTCTCTCAG*  *GCCCAAGATGCCCTTCAGT* |
